# Fluorescent amplified fragment length polymorphism (FAFLP) genotyping demonstrates the role of biofilm-producing methicillin-resistant periocular *Staphylococcus epidermidis *strains in postoperative endophthalmitis

**DOI:** 10.1186/1471-2415-6-1

**Published:** 2006-01-05

**Authors:** Prashanth Kenchappa, Aparna Duggirala, Niyaz Ahmed, Avinash Pathengay, Taraprasad Das, Seyed E Hasnain, Savitri Sharma

**Affiliations:** 1 Laboratory of Molecular and Cell biology, Centre for DNA Fingerprinting and Diagnostics (CDFD), Nacharam, Hyderabad – 500 076, India; 2 Jhaveri Microbiology Centre, L V Prasad Eye Institute, Banjara Hills, Hyderabad – 500 034, India; 3 Jawaharlal Nehru Centre for Advanced Scientific Research (JNCASR), Jakkur, Bangalore, India

## Abstract

**Background:**

An observational case series was used to study the virulence characteristics and genotypes of paired *Staphylococcus epidermidis *isolates cultured from intraocular samples and from periocular environment of patients with postcataract surgery endophthalmitis.

**Methods:**

Eight *S. epidermidis *isolates were obtained from three patients (2 from patients #1 and 2 and 4 from patient #3) whose vitreous and/or anterior chamber (AC) specimens and preoperative lid/conjunctiva samples were culture positive. Cultures were identified by API-Staph phenotypic identification system and genotypically characterized by Fluorescent Amplified Fragment Length Polymorphism (FAFLP) and checked for their antimicrobial susceptibility. The isolates were tested for biofilm-production and methicillin-resistance (MR) by PCR amplification of *icaAB *and *mecA *gene respectively.

**Results:**

Four out of eight *S. epidermidis *strains showed multiple drug resistance (MDR). All the eight strains were PCR positive for *mecA *gene whereas seven out of eight strains were positive for *icaAB *genes. In all three patients FAFLP typing established vitreous isolates of *S. epidermidis *strains to be indistinguishable from the strains isolated from the patient's conjunctival swabs. However, from patient number three there was one isolate (1030b from lid swab), which appeared to be nonpathogenic and ancestral having minor but significant differences from other three strains from the same patient. This strain also lacked *icaAB *gene. *In silico *analysis indicated possible evolution of other strains from this strain in the patient.

**Conclusion:**

Methicillin-resistant biofilm positive *S. epidermidis *strains colonizing the conjunctiva and eyelid were responsible for postoperative endophthalmitis (POE).

## Background

Endophthalmitis is an important ocular disease that is most commonly caused by postoperative and posttraumatic introduction of bacteria into the posterior segment of the eye. In severe infections, visual acuity is greatly diminished or completely lost [1]. Indigenous ocular flora is presumed to be a source of infectious organisms in bacterial postoperative endophthalmitis (POE). Bacteria have been recovered from the anterior chamber (AC) during the cataract wound closure in many cases [2]. A few studies have examined the genetic relationship between organisms isolated from the eyelid/conjunctiva, and organisms recovered from the vitreous of patients with POE [3,4].

Coagulase-negative staphylococci (CoNS) have been reported as the most frequent cause of bacterial POE [5]. Among CoNS, *S. epidermidis *seems to be predominantly present as normal flora of the lids and conjunctiva and implicated as a source of infection [1,3,4]. Based on the current literature, one may hypothesize that the commensal organisms turn virulent either prior to or once they gain intraocular access. With reference to intraocular infections, the transformation of commensal bacteria into virulent forms has not been studied at molecular level. Our aim was to investigate the genetic characteristics in strains isolated from extraocular samples and intraocular specimens and determine the variations, if any. We analyzed paired *S. epidermidis *isolates from an individual patient's eyelid/conjunctiva and vitreous/AC specimen by using FAFLP, a recently developed potential molecular strains-typing technique. In addition, we attempted to detect genes such as *icaAB*, *mecA *that might enhance virulence among the isolates by virtue of their role in biofilm formation, invasiveness and development of resistance to antibiotics.

## Methods

We examined 10 cases of POE after cataract surgery. All cases were referred to L. V. Prasad eye institute for management. Samples were collected from the eyelids and conjunctiva using cotton swabs from patients scheduled to undergo pars plana vitrectomy and intraocular antibiotic injection for the management of endophthalmitis. During surgery AC fluid and/or vitreous biopsy was collected from all cases. While the swabs were inoculated onto blood agar and brain heart infusion broth, the intraocular fluids were processed for bacteria and fungi as described earlier [6]. A total of eight isolates of *S. epidermidis *from three patients were included in the study (Table 1).

**Table 1:** Clinical characteristics of the patients having POE with details of strains and their origin. All patients underwent phacoemulsification with intraocular lens (IOL) implantation.

Patient No.	Age/Sex	Predisposing factors/Systemic disease	Site of isolation	Strain ID	Species	Antibiotype^a^	*mecA*/*icaAB*	Visual Acuity
1	43/M	Eye lid swelling	AC fluid	L778a/03	*S.epidermidis*	Oxa, G	+/+	20/60
			Eye lid	L778b/03	*S.epidermidis*	Oxa, G	+/+	
2	60/M	Hypertension	Vitreous	L849a/03	*S.epidermidis*	Oxa	+/+	20/25
			Eye lid	L849b/03	*S.epidermidis*	Oxa	+/+	
3	58/F	Diabetic retinopathy	Vitreous	L1030a/03	*S.epidermidis*	Ak, Ca, Cip, G, Oxa	+/+	20/40
			Eye lid	L1030b/03	*S.epidermidis*	Ak, Ca, Cip, G, Oxa	+/-	
			AC fluid	L1030c/03	*S.epidermidis*	Ak, Ca, Cip, G, Oxa	+/+	
			Conjunctiva	L1030d/03	*S.epidermidis*	Ak, Ca, Cip, G, Oxa	+/+	

^a ^Antibiotic resistance; Oxa – Oxacillin; G – Gentamicin; Ak – Amikacin; Ca – Ceftazidime; Cip – Ciprofloxacin.

The organisms were identified by using API Staph identification system (bioMerieux, France). Sensitivity to several antibiotics namely amikacin, ceftazidime, ciprofloxacin, gentamicin, and oxacillin was determined by Kirby Bauer disk diffusion method. Genomic DNA was isolated from all the eight strains according to methods described earlier [7]. PCR primers and conditions for amplification of *icaA *and *icaB *genes were as described by Frebourg et al [8]. Strains were checked for the presence of *mecA *by PCR that corresponds to unique penicillin-binding protein (PBP2a), directly associated with methicillin resistance (MR). All the strains were subjected to FAFLP by using *EcoRI*+0 and *MseI*+A primer combinations for selective PCR. FAFLP experiment and analysis (PE Biosystems, UK) were done as per the manufacturer's instructions. FAFLP electropherograms were analyzed using Genescan 3.7 and Genotyper 3.7 softwares (PE Biosystems) as described earlier [9]. The percentage similarities/differences between the patterns generated by different strains were calculated using the Dice correlation coefficient. A dendrogram representing the similarity coefficients was constructed using neighbor joining method [9].

## Results

**Figure 1:**
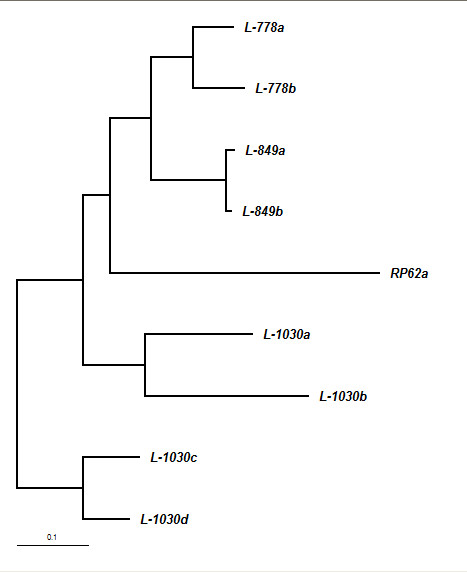
Neighbor joining tree showing similarity levels deduced from the genotyper data derived from FAFLP profiles of all the eight strains isolated from three patients along with the reference strain RP62a.

**Figure 2:**
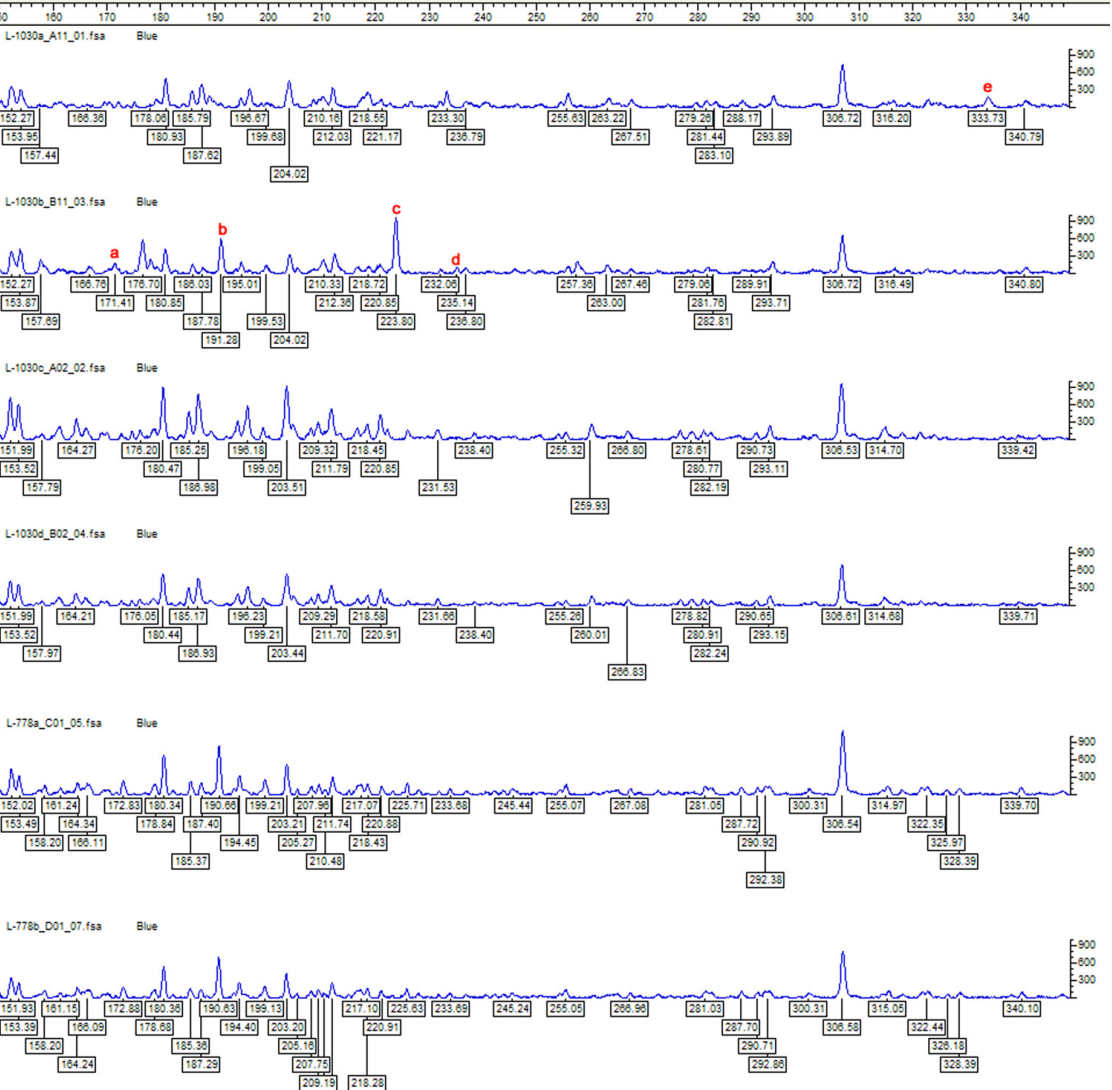
*S. epidermidis *strain specific FAFLP electropherograms depicting the range between 150–350 bp for *Mse*I+A selectivity tested, showing number and fragment sizes as well as the peak heights. The genotyper plots of paired isolates from patient 1 and patient 3. FAFLP patterns in order (top to bottom) are L-1030a, L-1030b, L-1030c, L-1030d, L-778a, and L-778b. Polymorphic fragment in L-1030a designated as 'e' in the figure (333 bp); polymorphic fragments in L-1030b designated as a, b, c, d (171, 191, 223, 235 bp).

Table 1 shows the clinical characteristics of all three cases included in the study. Paired strains obtained from each of these patients showed identical antibiotic sensitivity profile. Six out of eight strains were resistant to more than one antibiotic tested. MR was demonstrated in all the eight strains with the amplification of *mecA*. Seven out of eight strains showed *icaAB *gene amplification specific for biofilm production. FAFLP profiles revealed that intraocular *S. epidermidis *were indistinguishable from the conjunctival/lid isolates in all three patients (having < 1 % genetic distance among the paired strains)(figure 1). Three of the four strains from the third patient were identical and clonally originating, while the fourth strain (1030b) had minor differences from the rest in having four polymorphic fragments (figure 2). This strain was also *icaAB *negative. 1030b strain showed closer similarity with 1030a (< 3% genetic distance) and clustered with it in dendrogram (figure 1). Thus, we believe that in the third patient, POE was caused by strains that were clonally evolving and having minor genetic variations.

## Discussion

*S. epidermidis *present in the patient's periocular flora plays a significant role in causing POE [1,2]. This has been proven by a few studies using pulse field gel electrophoresis (PFGE) technique [2-4]. Application of advanced genotyping tools for typing of bacteria can give useful information regarding species distribution in periocular surfaces as well as help in understanding transmission dynamics and sources of infection, besides being useful in determining strain diversity with precision [4,7]. FAFLP typing in the present investigation established vitreous isolates of *S. epidermidis *strains were indistinguishable from the strains isolated from the patient's conjunctival swabs in all the 3 cases. These findings underscore the significant role of periocular flora in causation of POE. In this study, we examined the two important virulence factors of *S epidermidis *namely the biofilm production and multidrug resistance (MDR) of the strains associated with POE [8]. Biofilm- formation (BF) is one of the factors associated with increased virulence wherein *icaAB *genes were detected significantly more in infecting strains rather than in commensals [8]. MDR strains are known to cause greater intraocular inflammation than the susceptible strains in animal models [10] implying that these strains are more virulent. MR *S epidermidis *(MRSE) causing POE is difficult to treat. In recent years, the susceptibility of *S. epidermidis *has changed dramatically wherein nearly half of the strains are MDR. In particular, MR is frequent among *S. epidermidis *strains on a global scale [10] and the findings were similar in this study. In the present study, all the strains were MR, seven out of eight strains were positive for BF which is striking and substantiates earlier findings [8,10]. Presence of local/systemic risk factors and prophylactic antibiotic may enhance the role of MDR Coagulase negative Staphylococci in causing POE. One of our patients (no. 3) was diabetic for past 10 years, which is a possible risk factor. Information about prior antibiotic usage in our patients was not available. i*caAB *gene was detected in seven strains in this study indicating biofilm positivity. Strain 1030b appear to be ancestral with the absence of *icaAB*. 1030b generated four polymorphic bands (171, 191, 223, 235 bp) in FAFLP that were lacking in other three strains obtained from the same patient (figure 2) and this was the only *icaAB *negative strain. This finding suggests that biofilm-producing clones of resistant *S. epidermidis *were clonally evolving with changes occurring in their genomes. These clones appear to have genetic alteration that possibly indicates enhanced ability to cause POE. Strain 1030b from the lid of patient 3 is most likely nonpathogenic and ancient while other three strains from the same patient might have evolved from 1030b into more virulent types.

Predictive insilico AFLP methods with *EcoRI*+0 and *MseI*+A selectivity used on sequenced genomes of two *S. epidermidis *strains that are in the public domain and their extrapolation and comparison with our data showed interesting results. 1030b strain showed differential amplification of four genomic regions whereas 1030a, 1030c, 1030d lacked amplification of these regions which might be due to the mutation {insertion and deletion (Indels)} in *EcoRI *and *MseI *restriction site sequences. These corresponding polymorphisms are mapped to ORFs such as SE0884, SE0177, SE2068, SE0841 in ATCC 12228 and SE0775, SE2397, SE 2081, SE1995 in RP62a strain. Modification in at least two of the above ORFs namely SE0775 (fbe gene) and SE2081 putatively coding for fibronectin/fibrinogen binding protein and an adhesion protein respectively, seems to have definite role in increasing virulence. One recent study has showed that Fbe is a major factor involved in adherence of *S. epidermidis *to fibrinogen [11]. Antibodies against Fbe could block adherence of the bacteria to fibrinogen-coated surfaces. SE2081 is putative adhesion lipoprotein that may also have role in adhesion. Mutations in these two ORFs in the genomes of 1030a, 1030c, and 1030d might have enhanced their virulence favoring their survival under stressed condition.

## Conclusion

Refined molecular typing tool like FAFLP potentially presents as a highly sensitive rapid technique for strain typing. Methicillin-resistant biofilm positive *S. epidermidis *strains colonizing the conjunctiva and eyelid may be responsible for POE. The versatile results of our study show that FAFLP can detect subtle genetic variations in the colonizing strains that may explain their possible role in altered virulence.

## List of abbreviations used

AC: Anterior chamber

POE: Postoperative endophthalmitis

CoNS: Coagulase negative staphylococci

FAFLP: Fluorescence amplified fragment length polymorphisms

MDR: Multiple Drug Resistance

MR: Methicillin resistance

BF: Biofilm formation

MRSE: Methicillin Resistant *Staphylococcus epidermidis*

PFGE: Pulse Field Gel Electrophoresis

IOL: Intraocular lens

## Competing interests

The author(s) declare that they have no competing interests

## Authors' contributions

**PK: **Conception and design, performed FAFLP experiments, analysis and interpretation, writing the article, provision of materials, patients, or resources, obtaining funding.

**AD: **Data collection, provision of materials, patients, or resources, literature search

**NA: **Analysis and interpretation, technical, or logistic support

**AP**: Data collection, provision of materials, patients, or resources

**TD: **Critical revision of the article, administrative, technical, or logistic support

**SEH: **Critical revision of the article, final approval of the article

**SS: **Conception and design, analysis and interpretation, writing the article, provision of materials, patients, or resources

All authors have read and approved the final manuscript.

## Pre-publication history

The pre-publication history for this paper can be accessed here:


